# Fatty Acid-Binding Protein 4 is Essential for the Inflammatory and Metabolic Response of Microglia to Lipopolysaccharide

**DOI:** 10.1007/s11481-023-10079-6

**Published:** 2023-08-09

**Authors:** Yoshiteru Kagawa, Yi Ling Low, Jae Pyun, Umberto Doglione, Jennifer L. Short, Yijun Pan, Joseph A. Nicolazzo

**Affiliations:** 1 Drug Delivery, Disposition and Dynamics, Monash Institute of Pharmaceutical Sciences, Monash University, 3052 Parkville, VIC Australia; 2 Department of Organ Anatomy, Tohoku University Graduate School of Medicine, Sendai, 980-8575 Japan; 3 Drug Discovery Biology, Monash Institute of Pharmaceutical Sciences, Monash University, 3052 Parkville, VIC Australia

**Keywords:** Fatty acid-binding protein 4, Microglia, Immunometabolism, Neuroinflammation, Uncoupling protein 2

## Abstract

Prolonged activation of microglia leads to excessive release of proinflammatory mediators, which are detrimental to brain health. Therefore, there are significant efforts to identify pathways mediating microglial activation. Recent studies have demonstrated that fatty acid-binding protein 4 (FABP4), a lipid binding protein, is a critical player in macrophage–mediated inflammation. Given that we have previously identified FABP4 in microglia, the aim of this study was to assess whether FABP4 activity contributed to inflammation, metabolism and immune function (i.e. immunometabolism) in immortalised mouse microglia (BV-2 cells) using the proinflammatory stimulus lipopolysaccharide (LPS) to induce general microglial activation. Microglial FABP4 expression was significantly increased following exposure to LPS, an outcome associated with a significant increase in microglial proliferation rate. LPS-stimulated BV-2 microglia demonstrated a significant increase in the production of reactive oxygen species (ROS) and tumour necrosis factor-alpha (TNF-α), phosphorylation of c-Jun N-terminal kinase (JNK), increased expression of Toll-like receptor 4 (TLR4), and reduced expression of uncoupling protein 2 (UCP2), all of which were reversed following FABP4 genetic silencing and chemical inhibition with BMS309403. The oxidation rate of ^3^H-oleic acid and microglial uptake of ^3^H-2-deoxy-D-glucose were modulated with LPS activation, processes which were restored with genetic and chemical inhibition of FABP4. This is the first study to report on the critical role of FABP4 in mediating the deleterious effects of LPS on microglial immunometabolism, suggesting that FABP4 may present as a novel therapeutic target to alleviate microglia-mediated neuroinflammation, a commonly reported factor in multiple neurodegenerative diseases.

**Graphical Abstract:**

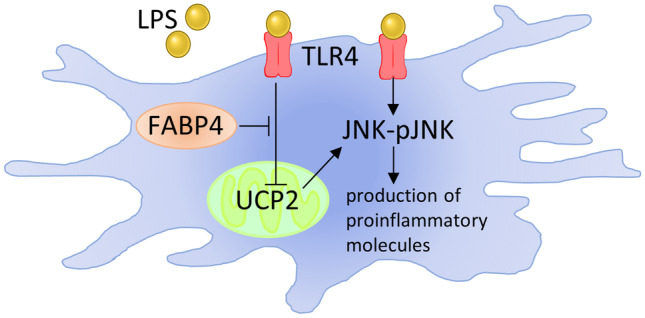

## Introduction

Neuroinflammation is defined as an inflammatory response in the central nervous system (CNS), and chronic neuroinflammation is commonly associated with the pathology of several neurodegenerative diseases, such as Alzheimer’s disease (AD), Parkinson’s disease (PD), and motor neuron disease (MND) (Subhramanyam et al. 2019). Neuroinflammation is characterised by excessive production and release of reactive oxygen species (ROS) and proinflammatory molecules, mainly by microglia, the resident immune cells of the CNS (Subhramanyam et al. 2019). When activated by a stimulus, microglia can adopt a spectrum of pro- or anti-inflammatory phenotypes in order to maintain CNS homeostasis. With prolonged proinflammatory microglia activation, excessive release of interleukin-1β (IL-1β), tumour necrosis factor-alpha (TNF-α), ROS, and nitric oxide (NO) can lead to a severe reduction in neuronal number and function (Kaur et al. 2014; Mallard et al. 2019). Given that microglia are heavily involved in neuroinflammation in various neurodegenerative diseases, approaches to ameliorate the microglia-mediated neuroinflammation could lead to reduced neuronal loss and attenuate the progression of neurodegenerative diseases, such AD, PD, and MND.

A fatty acid-binding protein (FABP) isoform was recently found to be involved in the activation process in macrophages (Hui et al. 2010). The FABP family consists of 11 different isoforms and they are mainly involved in chaperoning fatty acids (FAs) into and around cells. For example, FABP3 traffics arachidonic acid in cardiac and skeletal muscle cells (Hanhoff et al. 2002), while FABP5 is involved in the trafficking of docosahexaenoic acid across brain endothelial cells (Pan et al. 2015; Pan et al. 2016). In addition to their role in cellular and FA trafficking, one particular isoform (FABP4) has been reported to be involved in the inflammatory process. Elevated FABP4 expression has been detected in macrophages upon a proinflammatory insult, and this was associated with increased TNF-α and IL-12 levels (Hotamisligil and Bernlohr 2015). Resistance to several inflammatory disorders has been demonstrated in FABP4-deficient mice or in mice dosed with FABP4 inhibitors. For example, genetic deletion of FABP4 not only protected mice against atherosclerosis, but also compromised the inflammatory response induced by macrophages (Furuhashi et al. 2007), suggesting that FABP4 plays a critical role in peripheral macrophage-mediated inflammation.

FABP4 has also been reported to be expressed in microglia (Duffy et al. 2017; Low et al. 2021) and shown to be important in regulating the immunophenotype of microglia in response to exposure to a saturated fatty acid. Recently, Duffy et al. demonstrated that the proinflammatory response induced by a high palmitic acid diet was attenuated in the hypothalamic tissues of FABP4 deficient mice (Duffy et al. 2017). They further highlighted a novel FABP4-uncoupling protein 2 (UCP2) axis. UCP2 has been shown to mediate energetic processes of microglial activation states, and it was demonstrated that a non-specific FABP inhibitor increased UCP2 expression, reducing palmitic acid-induced NO and ROS release (Duffy et al. 2017). Therefore, inhibition of microglial FABP4 may be considered beneficial for neuroinflammation as ROS production is the main process that drives the microglia-mediated proinflammatory response (Naik and Dixit 2011). Indeed, lipopolysaccharide (LPS), a well-known pro-inflammatory molecule, reduces UCP2 expression, resulting in microglia adopting a proinflammatory phenotype with increased release of proinflammatory molecules (De Simone et al. 2015). Other studies have also reported that LPS reduces UCP2 expression in both macrophages and microglia, leading to increased ROS production and increased release of proinflammatory cytokines (Bai et al. 2005; Emre et al. 2007; De Simone et al. 2015). Collectively, these findings demonstrate that the FABP4-UCP2 axis may be necessary in the microglial-mediated neuroinflammation induced by LPS.

UCP2 is also involved in cellular energy pathways, and it is known that microglia utilise various cellular energy pathways in different activation states (Ghosh et al. 2018; Aldana 2019). The expression of UCP2 is involved in the metabolic switch between oxidative phosphorylation (OXPHOS) and the glycolytic pathway, as observed in neurons and mouse embryonic fibroblasts (Emre and Nübel 2010). An increase in UCP2 expression is suggested to discourage glycolysis, and encourage the use of OXPHOS to generate energy in cells. For example, knockdown of UCP2 in macrophages was found to increase lactate production, indicating that there was an increase in energy production through the glycolytic pathway over the other main energy-generating OXPHOS pathway (Xu et al. 2015). This link between UCP2 activity and energy pathways is critical as microglia exhibit a transient modulation of OXPHOS or glycolysis-related genes depending on their state of activation (Lauro and Limatola 2020). In the resting and anti-inflammatory phenotype, microglia rely mainly on OXPHOS to generate adenosine triphosphate (ATP), while in the proinflammatory phenotype, microglia favour glycolysis as an energy source. For example, BV-2 cells and primary mouse microglia exposed to IL-4 (an anti-inflammatory cytokine) exhibit a decrease in glucose consumption and lactate production and an increase in oxygen consumption, basal respiration, and ATP production (i.e. the OXPHOS pathway) (Lauro and Limatola 2020). In contrast, LPS-stimulated microglia favour the glycolytic pathway as observed through a decrease in mitochondrial ATP production and an increase in lactate production (Kalsbeek et al. 2016; Aldana 2019; Lauro and Limatola 2020).

As UCP2 is involved in microglial metabolism, and the presence of FABP4 can affect UCP2 expression, manipulating the levels of these proteins has the potential to affect microglial metabolism and phenotype. The overall aim of this study, therefore, was to investigate if modulation of FABP4 was able to affect microglial metabolism, and therefore the release of proinflammatory molecules during an activated state (i.e. microglial immunometabolism) following exposure to LPS. Using an immortalised mouse microglial cell line (BV-2), this study first investigated the impact of genetically silencing FABP4 on microglial metabolism, cytokine release, and c-Jun-N-terminal kinase (JNK) phosphorylation following LPS activation. Subsequently, the impact of BMS309403, a potent FABP4 inhibitor, on immunometabolism in BV-2 cells and primary cultured microglia was assessed to ascertain whether FABP4 could represent an exploitable pharmacological target for reducing neuroinflammation.

## Materials and Methods

### Materials

Dulbecco’s phosphate buffered saline (PBS), fetal bovine serum (FBS), Pierce BCA Protein Assay kit, Griess Reagent Kit, Macrophage-SFM (1X) media, Pierce 20x TBS buffer, mouse TNF-α uncoated ELISA kit, and mouse IL-6 uncoated ELISA kit were all purchased from Thermo Fisher Scientific (Grand Island, NY). Dulbecco’s Modified Eagle’s Medium F-12 Ham (DMEM/F-12), trypsin, trypan blue, bromophenol blue, β-mercaptoethanol, dimethyl sulfoxide (DMSO), bovine serum albumin (BSA), sodium hydroxide (NaOH), Tween-20, thiazolyl blue tetrazolium bromide (MTT), penicillin/streptomycin, Roche cOmplete^TM^ Protease inhibitor cocktail tablets, PhosSTOP tablets, LPS from Escherichia coli O111:B4, and 2′,7′-dichlorofluorescin diacetate (DCF-DA) were all purchased from Sigma-Aldrich (St Louis, MO). MACS^®^ CD11b+ Mouse MicroBeads, MS Columns, and the Adult Mouse Brain Dissociation Kit were purchased from Miltenyi Biotec (Bergisch Gladbach, Germany). iTaq Universal Probes One-Step kit (containing 2x probes RT-PCR reaction mix, iScript reverse transcriptase, and nuclease-free water), 0.2 µm nitrocellulose membrane, 0.45 µm immuno-blot low fluorescence PVDF membrane, Precision Plus Stained protein ladder, Mini-PROTEAN TGX 4-15% and 4-20% precast gels, and extra thick blotting paper were purchased from BioRad (Hercules, CA). QIAshredder columns, RNeasy Mini Plus kit, HiPerfect transfection reagent, FlexiTube FABP4 siRNA (SI02695322), and AllStar Negative Control siRNA were purchased from Qiagen (Hilden, Germany). LI-COR Intercept^®^ blocking buffer, LI-COR donkey anti-rabbit antibody (680 nm), and primary β-actin antibody were purchased from Millennium Science (Melbourne, Victoria, Australia). Anti-FABP3 antibody (ab45966) and anti-FABP4 antibody (ab66682) were obtained from Abcam (Cambridge, United Kingdom). Anti-FABP5 antibody (39926), anti-UCP2 antibody (D105V), SAPK/JNK antibody (9252), and phospho-SAPK/JNK (Thr183/Tyr185) antibody (81E11) were purchased from Cell Signalling Technology (Danvers, MA). Anti-TLR4 (48-2300) antibody was purchased from Thermo Fisher Scientific and BMS309403 was purchased from Cayman Chemicals (Ann Arbor, MI). ^3^H-oleic acid (3H-OA) was purchased from American Radiolabelled Chemicals Inc (St. Louis, MO), while ^3^H-2-deoxy-D-glucose (^3^H-2-DG) and Ultima Gold liquid scintillation cocktail were purchased from Perkin-Elmer (Boston, MA).

### Culturing of BV-2 Cells and Primary Mouse Microglia

BV-2 cells, originating from the laboratory of Dr. Elisabetta Blasi (Blasi et al. 1990), were generously provided by Dr. Linda J. Van Eldik (Lexington, KY). BV-2 cells were cultured in DMEM/F-12 media supplemented with 10% (v/v) FBS and 1% (v/v) penicillin/streptomycin. Cells were seeded into required plates at 20,000 cells/cm^2^, which were left in the 37 °C, 5% CO_2_ incubator until they reached 80% confluency and were used for experiments.

All experiments requiring the use of mice for isolation of microglia were approved by Monash Institute of Pharmaceutical Sciences Animal Ethics Committee (MIPS.27467) and performed in accordance with the National Health and Medical Research Council of Australia guidelines for the care and use of animals for scientific purposes. Brains removed from anesthetised C57BL/6 female mice (6-8 weeks old) were digested into a single cell suspension using the Adult Brain Dissociation Kit (Miltenyi Biotec, Bergisch Gladbach, Germany) and Miltenyi Biotec gentleMACS™ Dissociator as per the manufacturer’s protocol. CD11b^+^ cells were then magnetically selected using the MACS^®^ MicroBeads and MS Columns (Miltenyi Biotec). The selected cells were seeded at 50,000/cm^2^ into required plates and maintained in Macrophage-SFM media containing 1% (v/v) penicillin/streptomycin in a 37 °C, 5% CO_2_ incubator until confluent.

### Cell Treatment

The day after seeding, BV-2 cells were treated with 1 µg/mL LPS for 24 h. For FABP siRNA silencing, BV-2 cells were transfected 4 h post seeding as per the protocol previously described (Low et al. 2021). In brief, BV-2 cells were transfected with either FABP siRNA or AllStar Negative Control (siCont) for 5 min. After that, the concentration of siRNA complexes was diluted to 5 nM with FBS-containing media and cells were incubated with the siRNA treatment for 24 h prior to LPS being added to the cells. For BMS309403 treatment, BV-2 cells were co-treated with LPS (1 µg/mL) and BMS309403 (50 µM in vehicle) or vehicle (0.1% (v/v) DMSO) 24 h post-seeding and incubated for 24 h prior to being used for experiments. To assess the impact of BMS309403 post-microglial activation, BMS309403 (50 µM in vehicle) or vehicle (0.1% (v/v) DMSO) was added to BV-2 cells that had already been treated with LPS (1 µg/mL) for 4 h. To assess the impact of FABP4 inhibition in primary mouse microglia, 50 µM BMS309403 or vehicle (0.1% (v/v) DMSO) was added for 2 h prior to the addition of LPS (0.1 µg/mL) and microglia were co-treated for 24 h prior to being used for experiments. To assess the phosphorylation activity of JNK and total level of JNK, BV-2 cells were treated with LPS (1 µg/mL) for 30 min with or without siRNA or BMS309403 treatments.

### Western Blotting (WB) for Quantifying Protein Expression of FABPs, TLR4, UCP2, and P-JNK/JNK in BV-2 Cells

Following treatment, protein levels of FABPs, TLR4, UCP2, and p-JNK/JNK in BV-2 cells were quantitatively assessed using WB. Post-treatment, BV-2 cells were washed with ice-cold PBS prior to being lysed using Pierce IP lysis buffer supplemented with Roche cOmplete^TM^ mini protease inhibitor. Following centrifugation at 13400 *xg* for 10 min at 4 °C, protein count was determined using the Pierce BCA Protein Assay kit. 10 μg of protein was loaded in a 5:1 ratio with 6x Laemmli loading buffer on a BioRad 4-20% acrylamide precast gel, where the gel was run at 200 volts for 55 min using a BioRad Mini-PROTEAN Tetra cell (Hercules, CA). Following electrophoresis, the gel, alongside two thick blotting papers and a 0.2 μm nitrocellulose membrane, was equilibrated in transfer buffer containing 20% (v/v) methanol for 30 min. The protein from the gel was then transferred onto the nitrocellulose membrane at 11 volts for 42 min using a BioRad semi-dry transblot (Hercules, CA). The membrane was incubated in LI-COR Intercept^®^ blocking buffer before being incubated overnight at 4 °C with primary antibodies to specific mouse FABP isoforms (1:5000 dilution), TLR4 (1:500 dilution) or UCP2 (1:1000 dilution) and anti-mouse primary β-actin (1:10000 dilution) diluted in LI-COR Intercept^®^ blocking buffer. The membrane was further incubated in donkey anti-rabbit secondary antibody (1:30000 dilution) for 2 h at room temperature. The membrane was imaged using an AmershamTM Typhoon scanner (Marlborough, MA). Densitometric analysis on WB bands was performed using Image J (Bethesda, MD), with the proteins of interest quantified relative to β-actin (housekeeping protein).

### Proliferation Assay and Cell Viability

The proliferation rate of BV-2 cells prepared in 3 different plates was investigated by simply counting the number of cells on a haemocytometer before and after LPS treatment for 12 and 24 hours. To ensure that the concentrations of BMS309403 used on BV-2 cells did not significantly affect cell viability, an MTT assay was performed. Post-treatment, BV-2 cells were washed once with PBS prior to adding 150 µL of 0.45 mg/mL MTT reagent (prepared in FBS-free DMEM/F-12 media) to each well. The plate was incubated at 37 °C with 5% CO_2_ for 4 h. After incubation, the MTT reagent was removed and 150 µL of DMSO was added into all wells for 30 min. The plate was immediately read on a Perkin-Elmer Enspire fluorescence plate reader (Boston, MA) at 540 nm. Following background subtraction, the percentage cell viability was expressed as a ratio of mean absorbance of BMS309403-treated cells over mean absorbance of vehicle-treated cells.

### Quantification of ^3^H-OA Oxidation Rate in BV-2 Cells

The oxidation rate of ^3^H-OA in BV-2 cells was measured as reported previously (Ma et al. 2020). Briefly, BV-2 cells were incubated with incubation medium containing 60 µM of OA and 1 μCi ^3^H-OA for 4 h at 37 °C with 5% CO_2_. Following incubation, a 100 μL aliquot of supernatant was removed and mixed with an equal volume of ice-cold 10% (v/v) trichloroacetic acid. The mixture was centrifuged at 13,000 *xg* for 5 min at 4 °C. A 150 μL aliquot of the resulting supernatant was then treated with cold methanol:chloroform (2:1) and 2 M KCl:HCl before being centrifuged at 3,000 *xg* for 5 min. An aliquot of the upper (aqueous) phase (containing the oxidised ^3^H-OA, ^3^H_2_O) was removed and mixed thoroughly with 2 mL of scintillation fluid (Ultima Gold cocktail) and the radioactivity was determined using a Perkin-Elmer 2800TR liquid scintillation counter (Boston, MA). Oxidised ^3^H-OA (^3^H_2_O) produced was normalised to total protein count (μg) using a BCA assay and reported as pmol/μg protein.

### Cellular Uptake of ^3^H-2-DG

BV-2 cells and primary mouse microglia were rinsed with blank media before being supplemented with 1 μCi of ^3^H-2-DG prepared in culture media. Uptake was ceased after 15 min by removing the ^3^H-2-DG and rinsing the cells with ice-cold PBS three times. The cells underwent a freeze-thaw cycle before being lysed using Pierce IP lysis buffer and mixed thoroughly with 2 mL of scintillation fluid (Ultima Gold cocktail) and the radioactivity was determined. The amount of ^3^H-2-DG in cells was normalised to total protein count (μg) using a BCA assay and reported as pmol/μg protein.

### Measurement of Intracellular ROS and NO Production

For the measurement of intracellular ROS, BV-2 cells were rinsed once with warm HBSS and then incubated with 40 µM of DCF-DA prepared in FBS-free media for 30 min at 37 °C in 5% CO_2_. Following incubation, the wells were rinsed once with warm HBSS and HBSS was added back into the wells before being read on a Perkin-Elmer Enspire plate reader (Perkin-Elmer) (λ_ex_: 488 nm, λ_em_: 535 nm).

For measurement of NO production, BV-2 cells were aliquoted into a 96-well plate, and the Griess assay was performed as per the manufacturer’s protocol (Molecular Probes, Eugene, OR). The plate was then read on a Perkin-Elmer Enspire plate reader at 548 nm. Nitrite concentrations in samples were back calculated from a standard curve prepared through serial dilution of NaNO_2_ prepared in media.

### Quantification of Proinflammatory Cytokine Levels Using ELISA

The ELISAs were performed as per the manufacturer’s protocol to measure the amount of proinflammatory cytokines (TNF-α and IL-6) released from BV-2 cells and primary mouse microglia following FABP4 knockdown and inhibition, in the presence and absence of LPS. Post-treatment, 100 μL of supernatant, alongside either 100 μL of TNF-α or IL-6 standards, was aliquoted into pre-coated ELISA plates. Following a 2 h incubation, the wells were washed with wash buffer (0.05% (v/v) Tween-20 in PBS) four times before 100 μL of detection antibody (specific to the cytokine of interest) was added into each well for 1 h. The wells were then washed four times with wash buffer before 100 μL of Streptavidin-HRP was added into each well for 30 min. The wells were again washed six times before the wells were incubated with 100 μL of TMB substrate for 15 min. Following the addition of 100 μL of Stop solution, the plates were immediately read on a Perkin-Elmer Enspire fluorescence plate reader at 450 nm and 570 nm. Absorbance values at 570 nm were subtracted from the values at 450 nm to account for background. The concentration of cytokines released was normalised to total protein count (mg) determined with a BCA assay and reported as pg/mg protein.

### Data and Statistical Analyses

Data analyses were performed using Graphpad Prism 8. All data were expressed as mean ± SEM, where all replicates performed were biological replicates. When comparing between two groups, a Student’s unpaired t-test was performed. When more than two groups were compared, an analysis of variance (ANOVA), followed by an appropriate post-hoc test, as defined in the figure legends, was performed. Values of p<0.05 were considered statistically significant.

## Results

### FABP4 is Involved in LPS-induced Microglial Activation

**Fig. 1:**
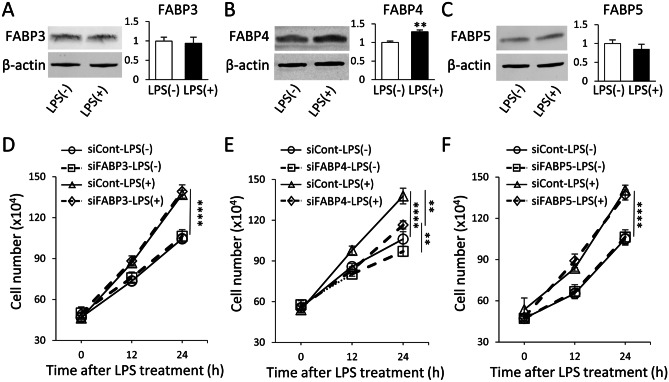
FABP4 is involved in LPS-induced microglial activation. **A, B, C** Protein expression of (A) FABP3, (B) FABP4, and (C) FABP5 in BV-2 cells normalised to β-actin with and without a 24 h treatment with 1 µg/mL LPS. Bar graphs show band density analysed with Image J software. Data are represented as mean ± SEM (n=6-7), where **p<0.01, as assessed using a Student’s unpaired t-test. **D, E, F** The proliferation rate of BV-2 cells treated with (D) FABP3 siRNA (siFABP3), (E) FABP4 siRNA (siFABP4), or (F) FABP5 siRNA (siFABP5) (5 nM, 24 h) relative to siCont in the presence and absence of 1 µg/mL LPS. Data are represented as mean ± SEM (n=3), where **p<0.01 and ****p<0.0001 as assessed using a two-way ANOVA, followed by a post-hoc Tukey’s test

The protein expression of FABP3, FABP4, and FABP5 (the three FABP isoforms present in BV-2 microglia) were first measured to assess if their expression was affected by LPS. Of the three FABP isoforms investigated, only FABP4 expression was significantly increased with 1 µg/mL LPS stimulation, while FABP3 and FABP5 remained unchanged (Fig. 1A-C).

In order to explore whether modulation of FABP expression by LPS correlated to microglial activation, the BV-2 cell proliferation rate was assessed in the presence and absence of FABP silencing as we demonstrated previously leads to an 82% reduction in FABP4 protein (Low et al. 2021). Without LPS treatment, the proliferation rate of BV-2 cells was not affected by each siFABP treatment (Fig. 1D-F). LPS treatment significantly increased BV-2 cellular proliferation rate, however only siRNA treatment to FABP4 had a suppressive effect on the proliferation rate compared to siCont, although there was still a significant difference in the proliferation rate of siFABP4-treated cells with and without LPS treatment (Fig. 1E). These results suggest that of the three isoforms assessed, only FABP4 has an important role in LPS induced microglial activation.

### FABP4 Silencing Decreased the LPS-induced Proinflammatory Response in BV-2 Cells

**Fig. 2:**
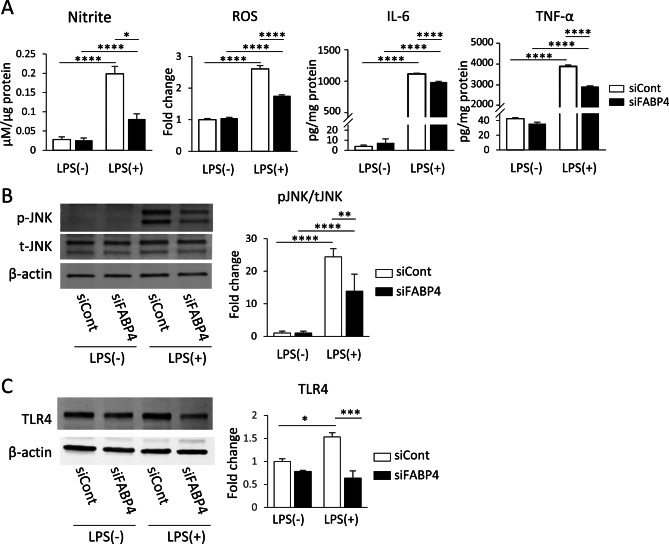
FABP4 silencing decreased the LPS-induced proinflammatory response in BV-2 cells. **A** The levels/concentrations of nitrite, ROS, IL-6, and TNF-α in BV-2 cells in the presence and absence of LPS with or without FABP4 siRNA (5 nM, 24 h). Data are presented as mean ± SEM (n = 6), where *p<0.05 and ****p<0.0001, as assessed using a two-way ANOVA, followed by a post-hoc Tukey’s test. **B** Western blot for pJNK and total tJNK in BV-2 cells with or without FABP4 siRNA, in the presence and absence of 1 μg/mL LPS for 30 min. The bar graph shows band density of pJNK/tJNK analysed with Image J software. Data are presented as mean ± SEM (n = 4), where **p<0.01 and ****p<0.0001, as assessed using a two-way ANOVA, followed by a post-hoc Tukey’s test. **C** Western blot for TLR4 and β-actin in BV-2 cells with or without FABP4 siRNA, in the presence and absence of 1 μg/mL LPS for 24 h. The bar graph shows band density of TLR4 normalised to β-actin analysed with Image J software. Data are presented as mean ± SEM (n = 3), where *p<0.05 and ***p<0.001, as assessed using a two-way ANOVA, followed by a post-hoc Tukey’s test

To assess the detailed role of FABP4 in microglial activation, we focused on the LPS-TLR4 signalling cascade. The levels of common proinflammatory molecules, NO, ROS, IL-6 and TNF-α in BV-2 cells were assessed following FABP4 silencing in vehicle and LPS-treated cells. LPS significantly increased the concentration of all proinflammatory molecules measured in BV-2 cells (Fig. 2A). Consistent with the proliferation rate, silencing of FABP4 significantly reduced the levels of proinflammatory molecules, albeit the levels of the proinflammatory molecules did not return to levels observed in vehicle-treated cells (Fig. 2A). As studies have demonstrated that mitogen-activated protein kinase (MAPK) signalling, including JNK, is activated following LPS stimulation, leading to an increase in the release of proinflammatory molecules (Kagawa et al. 2015), we assessed if FABP4 silencing was able to disrupt this pathway. Silencing of FABP4 attenuated the LPS-induced increase in pJNK although it did not return to the level observed in the vehicle-treated cells (Fig. 2B). We further assessed TLR4 expression and found FABP4 silencing attenuated the LPS-induced increase in TLR4 expression (Fig. 2C), suggesting that the proinflammatory action on the LPS-TLR4 signalling cascade is dependent on FABP4 in microglia.

### BMS309403 Reduces the LPS-induced Proinflammatory Response in BV-2 Cells

**Fig. 3:**
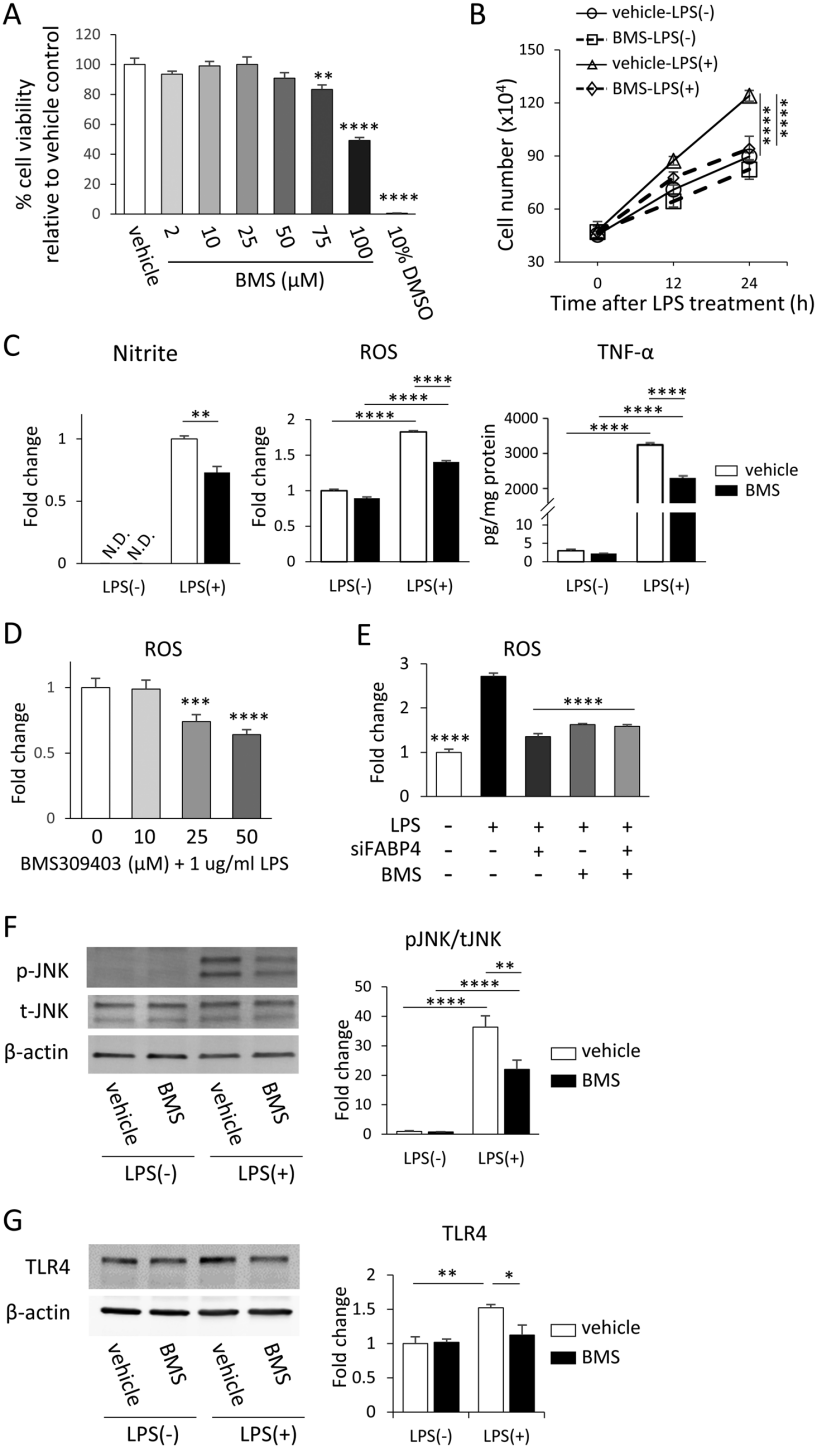
BMS309403 reduces the LPS-induced proinflammatory response in BV-2 cells. **A** BV-2 cell viability after being exposed to increasing concentrations of BMS309403 (2-100 μM) or vehicle for 24 h. 10% v/v DMSO served as a positive control. **p<0.01 and ****p<0.0001, as assessed using a one-way ANOVA followed by a post-hoc Dunnett’s test. **B** BV-2 cell proliferation rate at 12 and 24 h after 1 μg/mL LPS treatment. Prior to LPS treatment, cells were treated with vehicle or 50 μM BMS309403. Data are represented as mean ± SEM (n=3), where ****p<0.0001 as assessed using a two-way ANOVA followed by a post-hoc Tukey’s test. **C** The levels/concentrations of nitrite, ROS and TNF-α in BV-2 cells. Prior to LPS treatment, cells were treated with or without 50 μM BMS309403. Data are represented as mean ± SEM (n = 6) where **p<0.01, ****p<0.0001, as assessed using a two-way ANOVA followed by a post-hoc Tukey’s test. **D** ROS production in BV-2 cells following treatment with 10-50 µM BMS309403 and 1 µg/mL LPS for 24 hours. ***p<0.001, and ****p<0.0001, as assessed using a one-way ANOVA, followed by a post-hoc Dunnett’s test. **E** ROS production in BV-2 cells. Prior to LPS treatment, cells were treated with either 5 nM FABP4 siRNA, 50 μM BMS309403, or a combination of both. ****p<0.0001 as compared to the LPS-treated group, as assessed using a one-way ANOVA followed by a post-hoc Tukey’s test. All data above are represented as mean ± SEM (n = 6). **F** Western blot for pJNK and tJNK in BV-2 cells following BMS309403 treatment, in the presence and absence of 1 μg/mL LPS for 30 min. The bar graph shows band density of pJNK normalised to tJNK analysed with Image J software. Data are presented as mean ± SEM (n = 4), where **p<0.01 and ****p<0.0001, as assessed using a two-way ANOVA followed by a post-hoc Tukey’s test. **G** Western blot for TLR4 and β-actin in BV-2 cells following BMS309403 treatment, in the presence and absence of 1 μg/mL LPS for 24 h. The bar graph shows band density of TLR4 normalised to β-actin analysed with Image J software. Data are presented as mean ± SEM (n=3), where *p<0.05 and **p<0.01, as assessed using a two-way ANOVA followed by a post-hoc Tukey’s test

Given that changes in the microglial proinflammatory response were observed following genetic knockdown of FABP4, we investigated if FABP4 inhibition by BMS309403, a potent FABP4 chemical inhibitor, could affect the microglial response. An MTT assay performed following 24 h BMS309403 treatments demonstrated that 2-50 µM of BMS309403 did not significantly affect cell viability, while 75 µM and 100 µM BMS309403 resulted in a reduction in cell viability, as compared to vehicle control (Fig. 3A). Hence, a 50 μM concentration of BMS309403, for a treatment period of 24 h, was used in subsequent studies.

To determine the effect of BMS309403 on LPS-mediated microglial activity, the proliferation assay was performed. Following LPS treatment, the proliferation rate of BV-2 cells treated with BMS309403 was significantly decreased compared to the vehicle-treated cells (Fig. 3B). Similarly, BMS309403 attenuated the LPS-mediated increase in secretion of nitrite, ROS and TNF-α in BV-2 cells (Fig. 3C), in a concentration dependent manner as co-treatment of LPS with 25 µM and 50 µM BMS309403 resulted in a significant 0.26-fold and 0.36-fold reduction in ROS production, respectively, relative to the LPS-only treated group (Fig. 3D). To ensure that BMS309403 only acted on FABP4 to produce these anti-inflammatory effects, and not on other targets, the effects of BMS309403 were assessed in BV-2 cells in the presence and absence of FABP4 siRNA. BMS309403 was not able to further attenuate ROS levels any more in FABP4 siRNA treated cells relative to cells with FABP4 present (Fig. 3E), suggesting that the effects of BMS309403 on ROS were indeed due to FABP4 inhibition. Furthermore, the effect of BMS309403 on TLR4-LPS signalling was assessed and it was revealed that the LPS-mediated JNK activation and increase in TLR4 expression were attenuated by BMS309403 treatment (Fig. 3F, G).

### Genetic and Chemical Inhibition of FABP4 Reduced LPS-mediated and UCP2 Dependent Metabolic Changes in BV-2 Cells and Primary Mouse Microglia

**Fig. 4:**
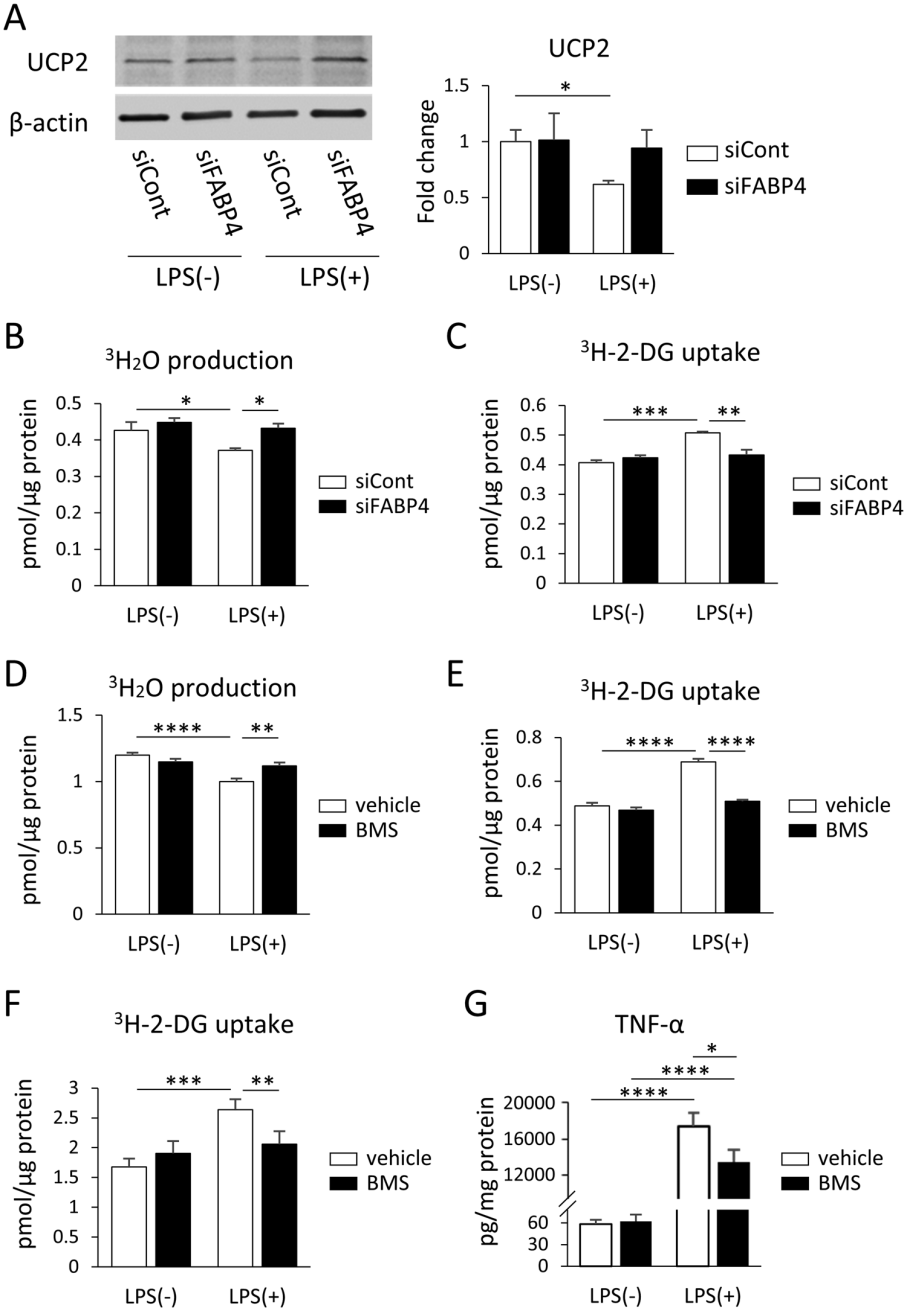
Genetic and chemical inhibition of FABP4 reduced LPS-mediated and UCP2 dependent metabolic changes in BV-2 cells and primary mouse microglia. **A** Western blot for UCP2 and β-actin in BV-2 cells with or without FABP4 siRNA (5 nM, 24 h), in the presence and absence of 1 μg/mL LPS for 30 min. The bar graph shows band density of UCP2/β-actin analysed with Image J software. Data are presented as mean ± SEM (n=3), where *p<0.05, as assessed using a two-way ANOVA followed by a post-hoc Tukey’s test. **B, C** ^3^H-OA oxidation (B) and ^3^H-2-DG uptake (C) in BV-2 cells following LPS treatment, with and without FABP4 genetic silencing. Data are represented as mean ± SEM (n=4-6), where *p<0.05, **p<0.01, and ***p<0.001, as assessed using a two-way ANOVA, followed by a post-hoc Tukey’s test. **D, E** ^3^H-OA oxidation (D) and ^3^H-2-DG uptake (E) in BV-2 cells following LPS treatment, with and without 50 µM BMS309403 treatment. Data are represented as mean ± SEM (n=4-6), where **p<0.01, and ****p<0.0001, as assessed using a two-way ANOVA followed by a post-hoc Tukey’s test. **F** ^3^H-OA oxidation in primary mouse microglia following 1 µg/mL LPS treatment for 24 h, with and without 50 µM BMS309403 treatment. Data are represented as mean ± SEM (n=8), where **p<0.01 and ***p<0.001 as assessed using two-way ANOVA followed by a post-hoc Tukey’s test. **G** The concentrations of TNF-α in primary mouse microglia treated with or without 0.1 µg/mL LPS for 24 h in the presence and absence of 50 µM BMS309403. Data are represented as mean ± SEM (n=8), where *p<0.05 and ****p<0.0001, as assessed using two-way ANOVA, followed by a post-hoc Tukey’s test

To examine how FABP4 regulates the LPS-induced proinflammatory cascade, the involvement of FABP4 in modulating UCP2 expression in LPS-treated BV-2 cells was assessed. UCP2 protein expression in BV-2 cells was significantly reduced when challenged with LPS, however when FABP4 in BV-2 cells was genetically silenced, the effect of LPS on UCP2 expression was negated, with UCP2 expression being restored back to the levels observed in siCont-treated cells (Fig. 4A). As UCP2 is involved in the metabolic switch between the OXPHOS and glycolysis pathway, we then examined the impact of genetically silencing FABP4 on microglial metabolism. Following LPS treatment, ^3^H-OA oxidation in BV-2 cells (which reflects the OXPHOS pathway) was significantly reduced (Fig. 4B), while ^3^H-2-DG uptake (which reflects glucose usage in the cell) was significantly increased (Fig. 4C), in line with the notion that inflammation leads to increased glucose utilisation. However, the effects of LPS on both ^3^H-OA oxidation and ^3^H-2-DG uptake levels were reversed when FABP4 was silenced (Fig. 4B, C), a result which was also observed with BMS309403 treatment (Fig. 4D, E), indicating that the FABP4-dependent UCP2 function regulates BV-2 cell energy metabolism. Furthermore, BMS309403 treatment on LPS-treated primary mouse cultured microglia restored ^3^H-2-DG uptake to levels measured in the absence of LPS (Fig. 4F) and this chemical inhibition of FABP4 also suppressed the LPS-mediated increase in TNF-α production in primary mouse microglia (Fig. 4G).

### BMS309403 Reduces ROS and TNF-α Production Post-microglial Activation

**Fig. 5:**
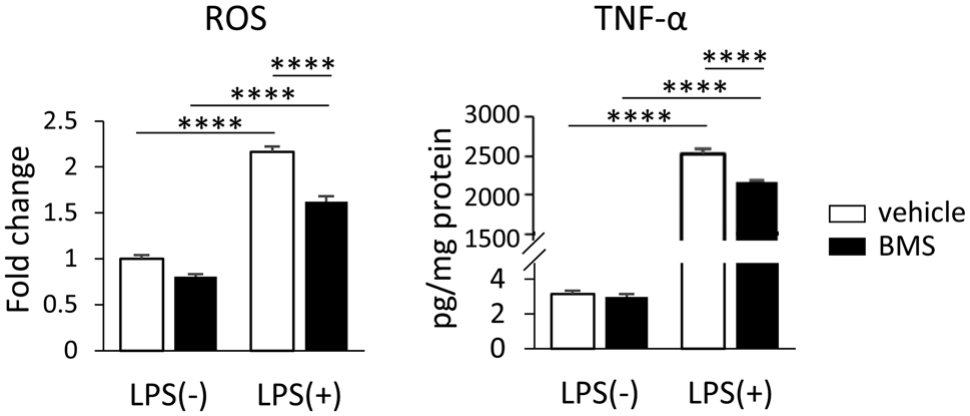
BMS309403 reduces ROS and TNF-α production post-microglial activation

To ascertain if BMS309403 could reduce inflammation following activation by LPS, in addition to the previous data showing BMS309403 could reduce LPS-mediated effects when exposed at the same time as LPS, BV-2 cells were treated with BMS309403 4 h after activation by LPS. When BMS309403 was introduced at this stage, it was still able to reduce LPS-mediated increases in ROS and TNF-α production (Fig. 5), suggesting that BMS309403 can reduce the proinflammatory phenotype even following microglial activation.

The levels/concentrations of ROS and TNF-α in BV-2 cells when exposed to 50 μM BMS309403 for 20 h after a 4 h pre-treatment with LPS (1 µg/mL). Data are represented as mean ± SEM (n=6), where ****p<0.0001, as assessed using a two-way ANOVA followed by a post-hoc Tukey’s test.

## Discussion

Both FABP4 and UCP2 have been reported to be involved in microglial metabolism, with the pathway that microglia employ to generate energy often reflecting their phenotype and activation state. Inhibition of FABP4 has been shown to lead to an increase in UCP2 expression in macrophages (Xu et al. 2015), which if occurs in microglia, would shift microglial metabolism towards OXPHOS rather than the glycolytic pathway for cellular energy generation. Such a change in microglial metabolism can ultimately alter microglia phenotype, thus resulting in changes to the release of pro- and anti-inflammatory molecules. Whilst it is known that there exists a link between FABP4 and microglial metabolism, it has yet to be investigated whether FABP4 mediates the deleterious effects of LPS on microglial activation and whether manipulating FABP4 expression would alter microglial metabolism and the release of proinflammatory molecules resulting from LPS insult. To address this, studies were first performed in BV-2 cells as they express similar phenotypical, phagocytic, and secretive functions as primary microglia (Henn et al. 2009). Several endpoint studies were then also performed in primary microglia isolated from adult C57BL/6 mice to confirm the role of FABP4 in LPS-mediated alterations to microglia immunometabolism in an additional microglial cell type.

Initially, studies were performed to assess if LPS affected the expression of the most prominent isoforms of FABPs in BV-2 cells, i.e. FABP3, FABP4, and FABP5 (Low et al. 2021). Out of the three isoforms assessed, only FABP4 protein expression was significantly increased by LPS exposure, which was expected, as elevated FABP4 expression has been reported during macrophage activation in several studies (Hui et al. 2010; Hotamisligil and Bernlohr 2015). Increased proliferation upon LPS treatment is one of the upregulated activities of microglia (Koss et al. 2019), thus we have examined how silencing of each FABP affected the BV-2 proliferation rate. FABP4 is involved in several metabolic pathways that are regulated by mitochondrial function, and for this reason, we assessed proliferation via simple counting of cells through a haemocytometer. When FABP4 was silenced, the LPS-mediated increase in proliferation rate was suppressed to the level of LPS-treated control cells (Fig. 1E), while there was no change in the proliferation rate in FABP3- and FABP5-silenced cells even under LPS stimulation (Fig. 1D, F). These results suggest that increased FABP4 expression upon LPS treatment might induce this increase in proliferation rate, with the LPS-mediated increase in proinflammatory molecule release from BV-2 cells being consistent with previous literature (Zhang et al. 2019; Kaewmool et al. 2020; Zhang et al. 2020). However, when FABP4 was silenced, a significant reduction in the TLR4-LPS signalling activity was observed, together with a reduction in proinflammatory molecule release, JNK signalling activity, and TLR4 expression (Fig. 2). While a significant reduction in the release of proinflammatory molecules was observed, the levels were not restored back to basal levels, which could be attributed to either an incomplete knockdown of FABP4 by siRNA or the other roles that FABP4 plays in the proinflammatory pathway. For example, it has been reported that FABP4 forms a positive feedback loop with JNK and activator protein-1 to trigger the inflammatory pathway in macrophages, where the presence of FABP4 enhances the release of TNF-α and IL-6 (Hui et al. 2010). Disruption of this feedback loop by inhibiting FABP4 attenuates part of the JNK activation and production of proinflammatory cytokines in macrophages (Hui et al. 2010). In our study, silencing of FABP4 in BV-2 cells could have potentially triggered the same effect, whereby the knockdown of FABP4 prevented the attenuation of the JNK-mediated inflammatory pathway, which resulted in the reduction, but not total ablation, of the release of proinflammatory molecules. Indeed, protein expression of pJNK in FABP4-silenced BV-2 cells was significantly reduced as compared to LPS-only treated cells, indicating that preventing the role of FABP4 in the JNK pathway likely resulted in the reduction of proinflammatory molecules observed. Interestingly, consistent with our results, a FABP4/NF-κB feedback loop has been reported to be involved in the Tat-induced microglial inflammatory response and genetic suppression of FABP4 was shown to attenuate the pro-inflammatory response, indicating that inhibition of FABP4 can be an effective therapeutic approach to suppress different intracellular signalling processes involved in inflammation (Zhou et al. 2022).

Given that our results supported genetic inhibition of FABP4 as a potential approach to regulate the LPS-mediated inflammatory response in BV-2 cells, we then investigated if FABP4 could be targeted through the use of a drug-like molecule. BMS309403 was chosen for this study as it is commonly used to reduce the function of FABP4 (Hui et al. 2010; Hoo et al. 2013), and the concentration chosen for this study has also been reported to significantly reduce FABP4 function in RAW 264.7 murine macrophage cells (Hui et al. 2010). The changes in microglia metabolism and the reduction in proinflammatory molecule release when BV-2 cells were treated with BMS309403 prior to LPS activation were similar to what was observed when FABP4 was genetically silenced in BV-2 cells, indicating that FABP4 can represent a pharmacological target to modulate the proinflammatory microglial phenotype. Furthermore, the significant reduction in pJNK expression following BMS309403 treatment suggested that inhibition of FABP4 could prevent the involvement of FABP4 in the positive feedback loop of the JNK pathway, thus reducing the release of proinflammatory molecules from BV-2 cells (Fig. 3). In addition, we also demonstrated that BV-2 cells co-treated with BMS309403 and FABP4 siRNA (which targets the FABP4 mRNA sequence directly) in the presence of LPS resulted in a similar fold reduction in ROS production as BMS309403 alone, indicating that the effect caused by BMS309403 was FABP4-specific, and not due to any off-target effects (Fig. 3E). Surprisingly, BMS309403 was also able to reduce the release of proinflammatory molecules following acute LPS stimulation (Fig. 5), further demonstrating that this compound can potentially be used to target FABP4 to modulate the proinflammatory microglia phenotype after inflammation has been initiated. Considering the anti-inflammatory effect of BMS309403 post-treatment with LPS, this FABP4 inhibitor may have the potential to reduce BV-2 cellular proliferation rate as both microglial proliferation as well as cytokine production are the phenotypes of activated microglia upon LPS stimulation, as reported previously (Koss et al. 2019). Although the inhibitor concentration of BMS309403 used was higher than its reported inhibitory constant (Ki) value (<2 nM) (Floresta et al. 2017), higher concentrations of BMS309403 have been used in multiple studies performed in macrophages (Furuhashi et al. 2007; Hui et al. 2010; Suhre et al. 2011). Furthermore, given that we did not observe any further effects of BMS309403 when BV-2 cells were co-treated with FABP4 siRNA, it suggests that the effects of BMS309403 at this concentration were indeed specific to FABP4. Most importantly, the results observed in BV-2 cells were able to be replicated in primary mouse microglia. LPS-activated primary microglia (with a purity of approximately 95%, as determined using FACs) responded to BMS309403 in a similar manner as activated BV-2 microglia in terms of microglial metabolism and TNF-α release (Fig. 4F, G). In previous studies, 1 μg/ml LPS has been used for the treatment of BV-2 cells (Li et al. 2023), while lower concentrations of LPS were used for the treatment of primary microglia cultures at concentrations ranging from 1 to 100 ng/ml (Neal et al. 2023; Tang et al. 2023; Li et al. 2022), suggesting that primary microglia may be more sensitive than BV-2 cells to the effects of LPS. In the current study, the treatment of primary microglia with 100 ng/ml LPS resulted in a significant increase in inflammatory marker expression, supporting that this concentration was ample to induce inflammation. In addition, while we did not assess the impact of co-treating BMS309403 with LPS on microglial response, we would anticipate that such a combination would lead to a similar result as observed with the 2 h BMS309403 pre-treatment.

While studies have reported the involvement of FABP4 and UCP2 in microglial metabolism, this is the first study to investigate the effect of knocking down FABP4 using siRNA on microglial metabolism in response to LPS. FABP4 silencing did not affect the expression of UCP2 in BV-2 cells without LPS treatment, while FABP4 silencing restored UCP2 levels back to those in vehicle-treated LPS-activated cells (Fig. 4A). Consistent with our results, So et al. have demonstrated no change to UCP2 expression in isolated microglia from WT and FABP4-deficient mice (So et al. 2022). Changes to UCP2 protein expression may not only be attributed to a transcriptional event, as it has been reported that its protein expression can also be regulated post-transcriptionally by several factors, including LPS and superoxide (Hass and Barnstable 2016). Given that UCP2 is an important regulator of microglial metabolism through mitochondrial function (Kim et al. 2019), it is reasonable that FABP4 silencing did not impact basal metabolism (as it did not impact UCP2 expression), but only changed microglial metabolism in the BV-2 cells with LPS stimulation (where UCP2 expression was modified). Indeed, both ^3^H-OA oxidation levels and ^3^H-2-DG uptake in LPS-activated cells were similar to levels of vehicle-treated cells when FABP4 was silenced (Fig. 4B, C), suggesting that microglia favoured OXPHOS over the glycolysis pathway to generate energy with FABP4 silencing. Since OXPHOS is able to generate more ATP relative to glycolysis, it may seem counterintuitive for activated proinflammatory microglia to utilise the glycolytic pathway as the main source of energy. However, the rate of glycolysis can be enhanced much faster than OXPHOS, as OXPHOS requires concomitant mitochondrial biogenesis (Amici et al. 2017). In addition, NO production from proinflammatory microglia also inhibits the OXPHOS pathway by inhibiting the electron transport chain, and therefore, proinflammatory microglia will favour the glycolytic pathway in order to generate the required energy in their highly energy demanding state (Orihuela et al. 2016). The restoration of ^3^H-OA oxidation levels in FABP4-silenced cells could also indicate normal electron transport chain function, suggesting that siRNA silencing of FABP4 might result in an attenuated release of proinflammatory molecules from activated microglia.

It is now largely recognised that microglial function is dependent on their pro-inflammatory (M1) or anti-inflammatory (M2) phenotype. M1 microglia release inflammatory mediators and induce inflammation and neurotoxicity, while M2 microglia release anti-inflammatory mediators and induce anti-inflammatory and neuroprotection (Guo et al. 2022). Recently, it has been reported that UCP2 in microglia functions as the master regulator of M1 and M2 microglial responses (De Simone et al. 2015; Kumar et al. 2022). For example, Simone et al. has demonstrated that LPS induced a decrease in UCP2 levels, which was paralleled by mitochondrial inner membrane potential depolarisation and increased mitochondrial ROS production (De Simone et al. 2015). On the other hand, the M2 stimulus IL-4 induces an increase in UCP2 levels and fails to induce M2 genes (mannose receptor 1 and IL-10) and to reduce M1 genes (iNOS and TNF-α) in UCP2-silenced microglia (De Simone et al. 2015). Taken together with our current results, it is suggested that UCP2 expression regulated by FABP4 might be involved in microglial M1/M2 polarization, which will be the subject of future studies.

Overall, the current study demonstrated that FABP4 is a potential therapeutic target in regulating microglial metabolism following LPS insult, which in turn can affect the release of proinflammatory molecules from microglia. While BMS309403 effectively inhibited FABP4 to alter microglia immunometabolism in the presence of a proinflammatory insult, its poor drug-like properties (e.g. high LogP) may potentially limit its use for therapeutic application. Therefore, identification of more drug-like FABP4 chemical inhibitors are required, which may then be used to alleviate microglia-induced neuroinflammation in many neurodegenerative diseases, such as AD, PD and MND.

## Data Availability

Not applicable.

## Abbreviations

2-DG: 2-deoxy-D-glucose
AD: Alzheimer’s disease
ATP: Adenosine triphosphate
FA: Fatty acid
FABP: Fatty acid-binding protein
IL: Interleukin
LPS: Lipopolysaccharide
MND: Motor neuron disease
NO: Nitric oxide
OA: Oleic acid
OXPHOS: Oxidative phosphorylation
PD: Parkinson’s disease
pJNK: Phospho-c-Jun N-terminal kinase
ROS: Reactive oxygen species
RT-qPCR: Quantitative reverse-transcriptase real-time polymerase chain reaction
siRNA: Small interfering RNA
TNF-α: Tumour necrosis factor-alpha
WB: Western blot/western blotting
